# Target-locked: A mechanism for disaggregase binding to aggregated proteins

**DOI:** 10.1016/j.jbc.2024.107165

**Published:** 2024-03-12

**Authors:** Trevor M. Morey, Walid A. Houry

**Affiliations:** 1 Department of Biochemistry, University of Toronto, Toronto, Ontario, Canada; 2 Department of Chemistry, University of Toronto, Toronto, Ontario, Canada

## Abstract

ClpG is a novel autonomous disaggregase found in *Pseudomonas aeruginosa* that confers resistance to lethal heat stress. The mechanism by which ClpG specifically targets protein aggregates for disaggregation is unknown. In their recent work published in JBC, Katikaridis *et al.* (2023) identify an avidity-based mechanism by which four or more ClpG subunits, through specific N-terminal hydrophobic residues located on an exposed β-sheet loop, interact with multiple hydrophobic patches on an aggregated protein substrate. This study establishes a model for substrate binding to a prokaryotic disaggregase that should inform further investigations into other autonomous disaggregases.

The maintenance of cellular protein homeostasis requires a fine-tuned balance between protein synthesis, folding, and degradation. Central to this process are molecular chaperones, a large class of proteins that collectively promote the folding of nascent polypeptides, shield proteins from unfolding stresses, disaggregate and refold misfolded and aggregated proteins, and target terminally misfolded proteins for degradation (1). In both prokaryotes and eukaryotes, some of the best studied molecular chaperones include members of the heat shock protein (HSP) family, such as DnaJ/Hsp40, DnaK/Hsp70, and HtpG/Hsp90, which primarily serve to fold nascent proteins into their native and active conformation (1). Alternatively, members of the Clp/Hsp100 family, including ClpX, ClpB/Hsp104, and ClpG, have been studied for their role in protein disaggregation, unfolding, refolding, and degradation (2, 3). Many of these Clp/Hsp100 chaperones belong to the AAA+ (ATPases associated with diverse cellular activities) superfamily of proteins that bind to substrates and unfold them through ATPase-driven activity (4). By interacting with various co-chaperones, the Clp/Hsp100 chaperones can either promote disaggregation of aggregated proteins (*e.g.* ClpB/DnaK) (2) or proteolysis of substrates (*e.g.* ClpX/ClpP) (3).

While our understanding of how the Hsp40/70/90 molecular chaperones recognize and promote the folding of nascent proteins has significantly increased over the past 20 years, the principles behind how Clp/Hsp100 disaggregase chaperones can target aggregated proteins, while sparing soluble, though unfolded, nascent proteins remains poorly characterized. In prokaryotes and fungi, ClpB/Hsp104 remains the canonical chaperone by which these organisms disaggregate protein aggregates (2). ClpB/Hsp104 has weak disaggregase activity when acting alone, requiring interaction with DnaK/Hsp70 to effectively target ClpB/Hsp104 to protein aggregates and to increase its unfolding activity (5). This contrasts with ClpG, a stand-alone autonomous disaggregase from *Pseudomonas aeruginosa* that has high aggregate unfolding activity independent of co-chaperones and has been shown by Katikaridis *et al.* (2021) to confer extreme thermotolerance to bacteria (6). While many of the Clp/Hsp100 family members share similarities in their AAA+ ATPase domains, they display variation in their N-terminal domains (NTDs), and current models suggest that substrate specificity of these disaggregases is regulated through these NTDs (7). Given the high basal ATPase activity of ClpG and its ability to specifically target protein aggregates without the need for co-chaperones, the mechanisms by which the NTD of ClpG regulates substrate specificity are of keen interest. Furthermore, gaining a clearer understanding of these mechanisms would be of great importance as members of the Clp/Hsp100 chaperones can both regulate thermotolerance in food-borne pathogens and increase virulency of highly infectious bacteria (8), making these chaperones potential antimicrobial targets.

To this end, Katikaridis *et al.* (2023) (9) recently sought to determine the mechanisms by which the unique NTD of *P. aeruginosa* ClpG regulates its specificity for protein aggregates. The authors utilized both biochemical and molecular approaches, as well as NMR spectroscopy, to characterize the interaction of the ClpG NTD with aggregated model substrates and to track the refolding ability of ClpG. Interestingly, the authors demonstrate that residues 1-71 of the ClpG NTD are not only critical but sufficient for aggregate targeting and refolding and, when fused to other AAA+ chaperones such as ClpB, can impart enhanced disaggregase activity independent of co-chaperones. Using NMR, the authors show that a set of conserved N-terminal Cys/His residues bind to and coordinate Zn^2+^, thus producing an extended antiparallel β-strand loop and forming a hydrophobic pocket with a nearby α-helix. Mutation of these residues reduced Zn^2+^ binding, abrogated ClpG disaggregase activity, and prevented enhancement of thermotolerance when mutant ClpG was expressed in *Escherichia coli*. Further characterization of the NMR structure revealed that residues Val^17^ and Leu^21^, located within the exposed β-strand loop, are essential for ClpG-mediated aggregate refolding. Lastly, by combining different ratios of active and NTD-truncated ClpG monomers, the authors conclude that aggregate targeting and subsequent disaggregase activity requires binding of a minimum of four ClpG NTDs within an active ClpG hexamer to a protein aggregate. Overall, the authors propose that disaggregation of protein aggregates by ClpG requires interaction between multiple ClpG NTDs and exposed hydrophobic patches on the substrate. Furthermore, the authors suggest that this feature of simultaneous docking would provide strong substrate specificity for larger protein aggregates, while sparing unfolded, though soluble, nascent polypeptides from the stand-alone disaggregase ClpG.

Mechanistically, this work not only establishes factors involved in targeting of protein aggregates by the autonomous disaggregase ClpG but also furthers our general understanding of how AAA+ disaggregases function. As noted by the authors, this mechanism of multi-subunit simultaneous docking to aggregated proteins has been observed for the canonical ClpB/DnaK disaggregase complex, though in this case, recruitment of ClpB to protein aggregates is indirect and first requires multiple DnaK monomers to bind (5). From a larger perspective, these results collectively suggest a model (Fig. 1) whereby nascent unfolded polypeptides are processed preferentially by interaction with DnaK/Hsp70 to produce folded and active proteins. Alternatively, in the case of aggregated misfolded proteins, multiple close-proximity and surface-exposed hydrophobic patches recruit disaggregase chaperones through multivalent interactions in a concentrated and localized manner, causing increased avidity for either ClpB (indirectly via increased DnaK binding) or ClpG (direct) (Fig. 1).

**Figure 1:**
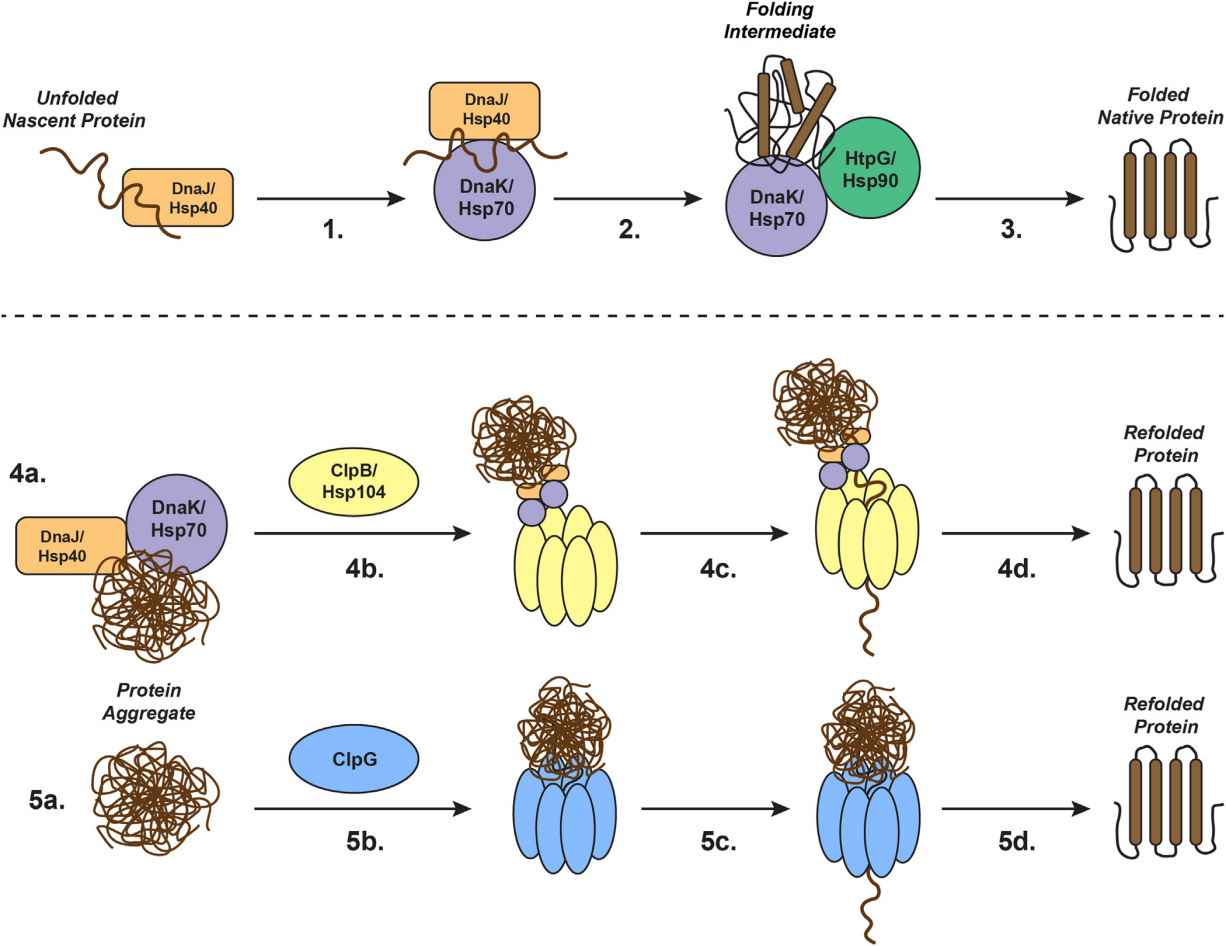
**Distinct mechanisms for the folding of nascent unfolded or aggregated proteins by members of the heat shock protein family of molecular chaperones.** Folding of nascent proteins begins by initial interaction of these unfolded polypeptides with DnaJ/Hsp40 and DnaK/Hsp70 (1). Following ATP hydrolysis (2), folding intermediates can be transferred to other HSPs, such as HtpG/Hsp90, which then fold these intermediates into their final folded and native state (3). Disaggregation of protein aggregates is facilitated by a subfamily of AAA+ Clp/Hsp100 chaperones, including ClpB/Hsp104 (4) or ClpG (5). Targeting of protein aggregates to ClpB/Hsp104 occurs in an indirect fashion, first requiring binding of multiple DnaJ/Hsp40 and DnaK/Hsp70 chaperones to exposed hydrophobic patches on aggregate surfaces (4a), which then recruits these aggregates to ClpB/Hsp104 hexamers (4b). Protein aggregates are then unfolded and threaded through a central pore in an ATP-dependent process (4c), resulting in release of a refolded substrate (4d). In contrast, aggregate targeting by ClpG (5a) occurs in an autonomous manner such that ClpG hexamers make multiple close-proximity and direct interactions with the hydrophobic surface of protein aggregates (5b). Subsequently, and following ATP hydrolysis (5c), protein aggregates are unfolded and threaded through a central pore similar to ClpB/Hsp104, resulting in release of refolded proteins (5d).

The proposed model of ClpG substrate recognition has exciting possibilities, both by providing a springboard for the study of other autonomous disaggregases in prokaryotes that may promote thermotolerance or virulence but also by serving as comparison to the study of orthologous Clp/Hsp100 members in eukaryotes, such as Hsp104 in yeast. Interestingly, metazoans do not express Hsp104, but recently, the ClpB/Hsp104-like Skd3 has been characterized in human cells as a novel protein disaggregase localized to the mitochondrial intermembrane space (10). In the future, it will be interesting to determine whether Skd3 or other eukaryotic disaggregases follow a similar aggregate-targeting mechanism as in prokaryotes.

## Conflict of interest

The authors declare that they have no conflicts of interest with the contents of this article.
